# Automated motion artifact detection in early pediatric diffusion MRI using a convolutional neural network

**DOI:** 10.1162/imag_a_00023

**Published:** 2023-10-17

**Authors:** Jayse Merle Weaver, Marissa DiPiero, Patrik Goncalves Rodrigues, Hassan Cordash, Richard J. Davidson, Elizabeth M. Planalp, Douglas C. Dean III

**Affiliations:** Department of Medical Physics, University of Wisconsin–Madison, Madison, WI, United States; Waisman Center, University of Wisconsin–Madison, Madison, WI, United States; Neuroscience Training Program, University of Wisconsin–Madison, Madison, WI, United States; Department of Psychology, University of Wisconsin–Madison, Madison, WI, United States; Center for Healthy Minds, University of Wisconsin–Madison, Madison WI, United States; Department of Psychiatry, University of Wisconsin–Madison, Madison, WI, United States; Department of Medicine, University of Wisconsin–Madison, Madison, WI, United States; Department of Pediatrics, University of Wisconsin–Madison, Madison, WI, United States

**Keywords:** diffusion weighted imaging, diffusion tensor imaging, quality control, pediatric neuroimaging, motion artifacts, convolutional neural network

## Abstract

Diffusion MRI (dMRI) is a widely used method to investigate the microstructure of the brain. Quality control (QC) of dMRI data is an important processing step that is performed prior to analysis using models such as diffusion tensor imaging (DTI) or neurite orientation dispersion and density imaging (NODDI). When processing dMRI data from infants and young children, where intra-scan motion is common, the identification and removal of motion artifacts is of the utmost importance. Manual QC of dMRI data is (1) time-consuming due to the large number of diffusion directions, (2) expensive, and (3) prone to subjective errors and observer variability. Prior techniques for automated dMRI QC have mostly been limited to adults or school-age children. Here, we propose a deep learning-based motion artifact detection tool for dMRI data acquired from infants and toddlers. The proposed framework uses a simple three-dimensional convolutional neural network (3DCNN) trained and tested on an early pediatric dataset of 2,276 dMRI volumes from 121 exams acquired at 1 month and 24 months of age. An average classification accuracy of 95% was achieved following four-fold cross-validation. A second dataset with different acquisition parameters and ages ranging from 2-36 months (consisting of 2,349 dMRI volumes from 26 exams) was used to test network generalizability, achieving 98% classification accuracy. Finally, to demonstrate the importance of motion artifact volume removal in a dMRI processing pipeline, the dMRI data were fit to the DTI and NODDI models and the parameter maps were compared with and without motion artifact removal.

## 1 Introduction

Diffusion magnetic resonance imaging (dMRI) has become one of the most widely utilized non-invasive imaging techniques for studying brain tissue microstructure in vivo. Sensitive to the movement of water molecules in biological tissues, which is in turn modulated by the density, spacing, and orientational organization of biological barriers (e.g., axons, dendrites, myelin), dMRI indirectly probes tissue microstructure and can be used to infer information about the brain’s structural architecture and connectivity (D. C. Alexander et al., 2019; DiPiero et al., 2023; Lebel & Deoni, 2018; Neil & Smyser, 2021; Ouyang et al., 2019; Tamnes et al., 2018). Moreover, the development of dMRI models, such as Diffusion Tensor Imaging (DTI; A. L. Alexander et al., 2007; Basser et al., 1994) and newer biophysical modeling techniques, including Neurite Orientation Dispersion and Density Imaging (NODDI; Zhang et al., 2012), provide unprecedented opportunities for quantitative investigation of the brain’s microstructure. To date, dMRI is used across clinical and biomedical research applications, including neurological and neurodegenerative disorders, such as Alzheimer’s Disease, multiple sclerosis, epilepsy (Goveas et al., 2015; Inglese & Bester, 2010; Lakhani et al., 2020; Yogarajah & Duncan, 2008); neuropsychiatric conditions, such as autism spectrum disorders and attention deficit and hyperactivity disorder (Aoki et al., 2018; Ismail et al., 2016; Kangarani-Farahani et al., 2022; Travers et al., 2012); brain tumors and ischemic stroke (Drake-Pérez et al., 2018); and characterizing microstructural changes during development and aging (Lebel & Deoni, 2018; MacDonald & Pike, 2021; Schilling et al., 2022). However, limitations of dMRI, particularly in pediatric populations, can pose significant challenges to processing, analysis, interpretation, and reliability of the quantitatively derived dMRI measures if not properly accounted for during pre-processing steps.

dMRI is prone to multiple types of artifacts, including subject motion, Eddy currents, and echo-planar imaging (EPI) related artifacts such as ghosting, chemical-shift, geometric distortions, and susceptibility (Jones & Cercignani, 2010; Le Bihan et al., 2006; Tax et al., 2022). Subject motion is especially problematic, and often unavoidable, when scanning challenging populations that may be unable to remain still for long periods of time, including infants and young children. Although children under 4 years of age who participate in research studies leveraging MRI often undergo imaging during natural, non-sedated sleep (Dean et al., 2014; Pollatou et al., 2022; Spann et al., 2022), artifact from motion remains a complicated issue. On the other hand, while scanning school-age children can often be performed while the child is awake, it frequently requires study staff to spend additional time acclimating the child to the MRI scanner environment and instructing them to remain still during the scan which can help reduce but not fully eliminate motion-related artifacts (De Bie et al., 2010; Dean et al., 2014; Pollatou et al., 2022; N. Raschle et al., 2012; N. M. Raschle et al., 2009; Spann et al., 2022). If motion-related artifacts are not corrected or accounted for prior to analysis, resulting signal errors and outliers may significantly influence derived dMRI measures and outcomes, including quantitative model-based parameters, diffusion tractography, and group comparisons (Chen et al., 2015; Le Bihan et al., 2006; Tax et al., 2022; Yendiki et al., 2014). For example, Yendiki et al. (2014) demonstrated that higher motion in an autism spectrum disorder (ASD) group led to decreases in fractional anisotropy (FA) and axial diffusivity (AD) and increases in radial diffusivity (RD). These parameter changes led to spurious group differences between the higher motion ASD group and the typically developing control group, with the severity of the motion artifact contributing to the extent of the group differences, as similar group differences were not seen in an ASD group with less motion (Yendiki et al., 2014). Additionally, the global nature of motion artifacts can cause voxel misalignment between volumes, leading to false higher ADC measurements at tissue boundaries due to partial volume effects (Le Bihan et al., 2006). Therefore, accurate identification of outliers (and other artifacts) in dMRI data is a necessary and critically important quality control (QC) step in any dMRI pre-processing pipeline to remove or account for potential sources of bias and variability as well as reduce unreliable results (Bammer et al., 2003; Reuter et al., 2015; Tax et al., 2022).

Currently, the gold standard for QC of dMRI data is manual visual inspection by a trained expert for identification of outliers in the dMRI signal within each individual diffusion volume. Most commonly when a volume is identified to contain artifacts, it is deemed unusable and is excluded from further processing and analysis. The manual QC of dMRI is extremely time-consuming as a large number of volumes are often acquired. Further, the manual QC process approaches impractical as large multi-center studies become much more prevalent; for example, ABCD (https://abcdstudy.org/; Casey et al., 2018; Jernigan et al., 2018; Volkow et al., 2018), HCP (https://www.humanconnectome.org/; Elam et al., 2021; Van Essen et al., 2013), dHCP (https://www.developingconnectome.org/; Edwards et al., 2022), BCP (https://babyconnectomeproject.org/; Howell et al., 2019), and HBCD (https://hbcdstudy.org/; Jordan et al., 2020), where hundreds-to-thousands of datasets are acquired each consisting of hundreds of dMRI volumes. Further, manual QC can be a subjective selection process. While the individuals performing the manual QC are trained experts, differences between raters could lead to different outcomes and higher variability in quality standards and outcome measures. Given the importance of QC to dMRI processing, there is an urgent need for the development of accurate automated methods to identify and either remove or account for artifact-corrupted data.

Several tools are currently in use for automated QC of dMRI data, including DTIPrep (Oguz et al., 2014), DTIStudio (Jiang et al., 2006), FSL EDDY and QUAD (Andersson & Sotiropoulos, 2016; Andersson et al., 2016; Bastiani et al., 2019; Jenkinson et al., 2012), and TORTOISE (Pierpaoli et al., 2010). These tools use differing approaches that aim to extract local image information and features that can be used to identify and, in some cases, retrospectively correct image outliers and artifacts. However, many of these tools have mixed performance in pediatric dMRI datasets compared to the performance from adult datasets, given an increased likelihood for large motion (Kelly et al., 2017). For example, the outlier slice detection and replacement tool within FSL EDDY reports poorer results when the slice outlier frequency is greater than 10% (Andersson et al., 2016), a common occurrence in pediatric datasets. It is unclear if implementing these motion correction techniques on cases with severe dropout has a negative effect on derived diffusion parameters such as those from DTI and NODDI. More recently, several deep learning (DL) approaches using convolutional neural networks (CNNs) have been proposed for automated QC of both structural (Fantini et al., 2018; Iglesias et al., 2017) and dMRI data (Ahmad et al., 2023; Ettehadi et al., 2021, 2022; Graham et al., 2018; Kelly et al., 2017; Samani et al., 2020). However, the training and validation datasets for these approaches were limited to neonates (<1 month of age) (Graham et al., 2018; Kelly et al., 2017) or school-age children and adults older than 6 years (Ahmad et al., 2023; Ettehadi et al., 2021, 2022; Samani et al., 2020). As such, there exists a need for automated QC of dMRI data tailored towards dMRI data from the early developmental years.

We propose a three-dimensional CNN (3DCNN) for motion artifact detection of multi-shell dMRI data acquired from pediatric subjects between the ages of 1 month and 35 months. The network is trained and cross-validated on a dataset composed of subjects scanned at 1 month and 24 months of age. Using an additional non-training dataset with different acquisition parameters and subject ages (2-35 months), we show that our network achieves similar performance on a dataset unseen during training despite the new acquisition parameter, brain sizes, and tissue contrasts. We evaluate the performance of our network by examining DTI- and NODDI-based measures derived from processing the dMRI data in three ways: (1) performing no QC; (2) manually removing motion-corrupted dMRI volumes; and (3) removing dMRI volumes based on our DL neural network labeling. Group and individual analyses of the DTI and NODDI measures fractional anisotropy (FA), radial diffusivity (RD), and intracellular volume fraction (ICVF) suggest high reproducibility between manual QC and DL model-based QC, and poor reproducibility between manual QC and the pipeline with no volume removal. These results demonstrate the proposed network can closely match the accuracy of manual QC on several early pediatric dMRI datasets, highlighting its effectiveness to identify motion-corrupted dMRI volumes that can significantly alter subsequently derived quantitative metrics, while saving time and avoiding the influence of potential subjective human error.

## 2 Materials and Methods

### 2.1 Overview

The proposed method contains a 3DCNN to detect motion artifacts in dMRI volumes acquired from pediatric subjects under 35 months of age. To evaluate the model’s performance and highlight the importance of identifying and removing corrupted volumes, DTI and NODDI parameters were obtained and compared using three different processing pipelines: (1) corrupted volumes manually labeled and removed, (2) corrupted volumes labeled by the model and removed, and (3) corrupted volumes are not removed. We first describe the two datasets that are used for training and testing the network. Then, the architecture, training, and optimization of our network are described. Finally, the processing pipeline and quantitative parameter analysis are discussed.

### 2.2 Datasets and curation

We utilized two infant dMRI datasets (herein referred to as Dataset A and B) acquired with different dMRI acquisition parameters from typically developing infant cohorts with different, but overlapping, age ranges. Both datasets were acquired with the approval of the University of Wisconsin-Madison Institutional Review Board, and written informed consent was obtained from the parents of each participating family. Dataset A contains longitudinal diffusion imaging data acquired at 1 and 24 months of age (Dean et al., 2017, 2021; Dean, Planalp, Wooten, Kecskemeti, et al., 2018; Dean, Planalp, Wooten, Schmidt, et al., 2018; Dowe et al., 2020; Planalp et al., 2023), while Dataset B was sourced from an ongoing longitudinal study in which participants are scanned up to three times approximately 6 months apart. All dMRI data were acquired during natural, non-sedated sleep (Dean et al., 2014). A summary of the demographics and acquisition parameters for each dataset can be found in Table 1.

**Table 1.** Dataset details and acquisition parameters.

Dataset	Unique subjects (male %)	Total diffusion acquisitions	Age range (mos.)	TR (ms)	TE (ms)	Resolution (mm)	b-values (s/mm^2^)[# of DWIs]	Total number of DWIs
A	116 (46.6%)	151	1, 24	8,400	94	2.0 × 2.0 × 2.0	0 [6] 350 [9] 800 [18] 1,500 [36]	69
B	17 (64.7%)	26	2-35	7,000	107	2.0 × 2.0 × 2.0	0 [6] 300 [9] 800 [15] 1,200 [30] 2,500 [45]	105

All exams used the same 3T scanner (Discovery MR 750, GE Healthcare, Waukesha, WI) and either an 8-channel (GE Healthcare) or a 32-channel (Nova Medical, Wakefield, MA) receive-only head coil.

**Fig. 1.**
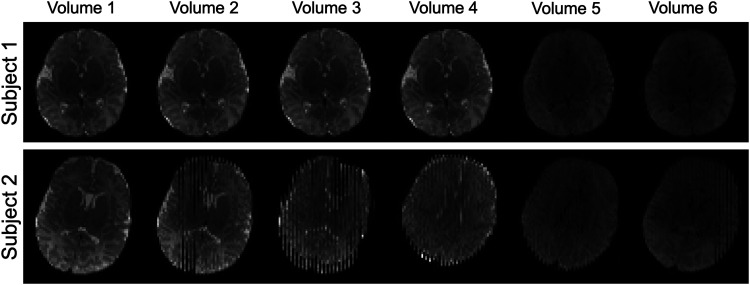
Representative diffusion encoding volumes from two 1-month-old subjects displaying a motion-free exam (Subject 1, top row) and an exam with numerous motion-corrupted volumes (Subject 2, bottom row).

Following image acquisition, manual quality control was performed by trained research staff members. Specifically, each individual diffusion-weighted volume was visually inspected for motion artifacts and annotated as either artifact-free or artifact. Following visual inspection and annotation, 11% of the 10,128 volumes in Dataset A and 5.3% of the 2,349 volumes in Dataset B were identified as motion corrupted. Figure 1 displays representative examples of volumes from 1-month-old subjects from Dataset A manually labeled either artifact-free or artifact. Following manual annotation, Dataset A was used to create the training and testing sets. To avoid problems that may arise from class imbalances, Dataset A was curated to maintain an equal class distribution with an identical number of motion-corrupted volumes and randomly selected normal volumes from each subject. In the case of more than half of a subject’s volumes being motion-corrupted, all volumes were included in the dataset. When the class imbalance in a dataset is high, DL algorithms may be biased towards the majority class which is especially problematic when correctly predicting the minority class is more important than the majority (Krawczyk, 2016). While algorithms can be modified to alleviate this majority bias, a data-level approach of balancing distributions was chosen due to the excess of motion-free volumes. Following data curation, Dataset A consisted of 2,276 volumes, of which approximately 50% were motion corrupted, from 121 acquisitions. Dataset B served as an additional unseen testing dataset, containing differing contrasts (due to age-related differences) and differences in b-values. All diffusion volumes were either zero-padded or cropped to a common size of 128 × 128 × 70, and voxel intensities for each volume were normalized to be between 0 and 1 to maintain the same scale across all subjects and b-values.

### 2.3 Model architecture and training

A 3D network was selected over a 2D network following considerations of technical limitations, ease of manual labeling, and interpretation. The memory limitations of GPUs often make whole-volume 3D approaches challenging, and prior studies have opted to use 2D architectures (Graham et al., 2018; Kelly et al., 2017; Samani et al., 2020) or 3D architectures that break up the full 3D volume into smaller slabs or ROIs (Ettehadi et al., 2021, 2022). However, whole-volume 3D approaches (Ahmad et al., 2023) are becoming more feasible with newer GPUs with increased memory capabilities. 3D approaches have the benefit of reducing the amount of time required for manual QC of a single subject, allowing for training datasets to contain a larger number of unique subjects. Additionally, artifacts may manifest differently in each plane, dependent on encoding directions. Thus, 2D approaches may need to consider each plane when curating data and training, leading to extra considerations when interpreting results such as a slice-count threshold for determining when an entire volume can be considered artifactual (Samani et al., 2020).

**Fig. 2.**
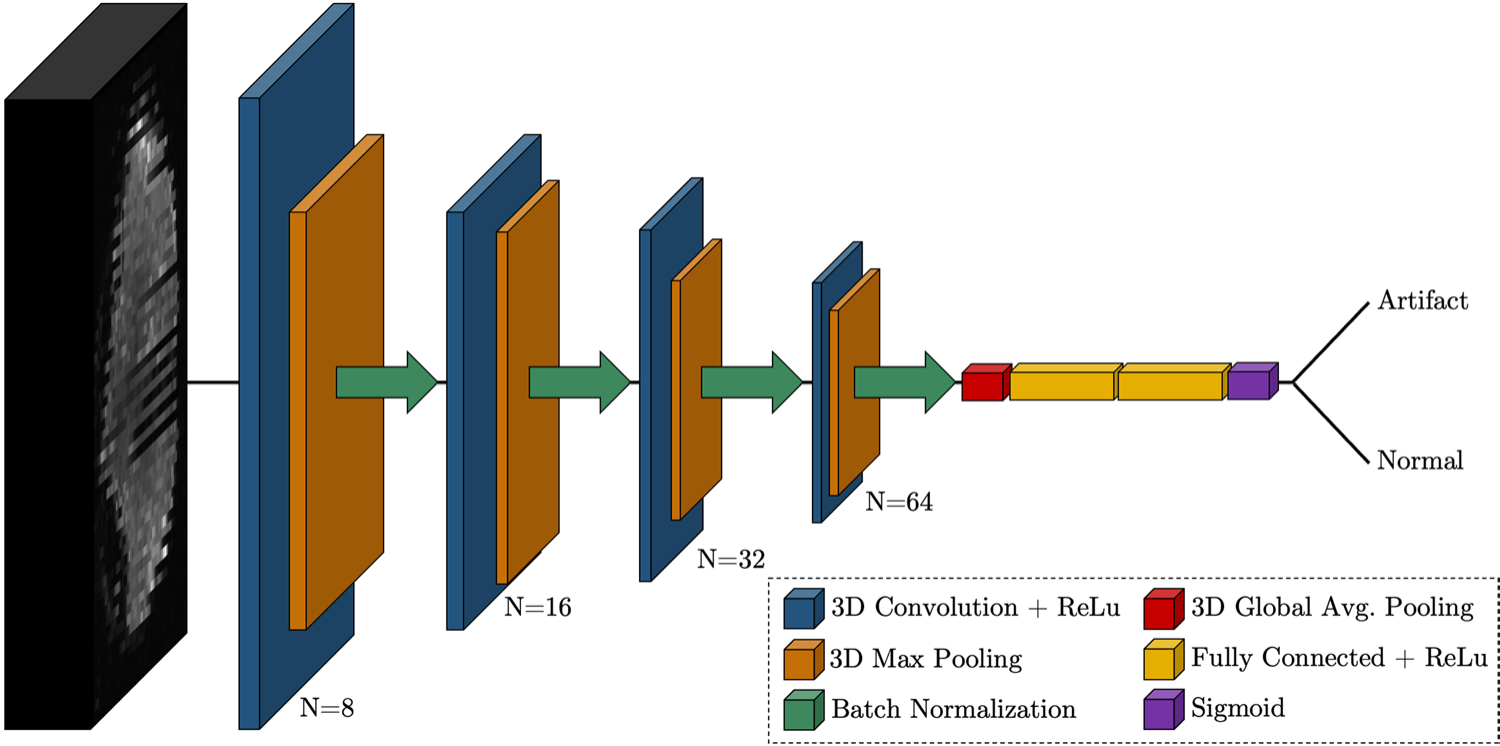
Proposed convolutional neural network architecture for the detection of motion artifacts in 3D diffusion MRI volumes of size 128 × 128 × 70. The network consists of four blocks of 3D convolution (N = number of filters), 3D max pooling (pool = 2), and batch normalization, followed by flattening, dense layers with 50% total dropout, and a sigmoid layer for final classification.

The 3DCNN architecture was implemented in Python 3.8 using Tensorflow 2.8.0 with Keras. An overview of the network architecture is depicted in Figure 2. The architecture is intentionally simple to both overcome the GPU memory challenges associated with 3D networks and avoid overfitting due to the relatively small number of training samples.

The network consists of four feature extraction blocks, with each block containing a 3D convolutional layer with a pool size of 3 × 3 × 3 and ReLU activation. Each convolutional layer is followed by a 3D max pooling layer with a stride of 2 and batch normalization. Within each block, the number of filters is increased (8, 16, 32, 64) as the input volume dimensions are reduced. The final output from the last block is flattened and passed to a fully connected layer with 128 neurons and a dropout rate of 50%. The output is passed to an additional fully connected layer with 128 neurons and then to a dense layer of 1 neuron with sigmoid activation appropriate for the binary classification problem.

Dataset A was used for training with a batch size of 8. Binary cross-entropy loss was minimized over 30 epochs using the Adam optimizer with an initial learning rate of 1 × 10^−3^ and learning rate decay. Training time was approximately 45 minutes utilizing a single GPU (NVIDIA RTX 2070) with 8 GB VRAM. K-fold cross-validation with k = 4 was used to test the model’s dependence on the input data, and the number of folds was chosen such that the testing datasets contained an acceptable number of both 1- and 24-month-old subjects after random shuffling. Finally, the folds were split based on subject rather than diffusion volumes to prevent data leakage that may occur when there is overlap between the training and testing sets (Tampu et al., 2022). If the data split was performed at a volume level, training and testing sets may each contain nearly identical b = 0 volumes or nonzero b-value volumes with similar directions from the same subject that may contain similar information and lead to inflated test statistics.

### 2.4 Validation

#### 2.4.1 Artifact prediction

The model was trained on Dataset A and evaluated on Datasets A and B using k-fold cross-validation with k = 4. For each of the training runs with a different fold left out as the test data, the output model was saved and used to predict labels for Dataset B. Due to the different brain volumes, contrasts, and b-values present in Dataset B, evaluation on Dataset B will provide a test of generalizability on unseen data for the trained network. Accuracy was used as the training metric, while precision, true positive rate (TPR), and true negative rate (TNR) were used for additional analysis. TPR is also referred to as recall for consistency with prior work. Each is defined below as:

$$Accuracy=\frac{TP+TN}{TP+TN+FP+FN}$$

$$Precision=\frac{TP}{TP+FP}$$

$$TruePositiveRate\left(Recall\right)=\frac{TP}{TP+FN}$$

$$TrueNegativeRate=\frac{TN}{TN+FP}$$

#### 2.4.2 Preprocessing and analysis

The model with the highest accuracy across both datasets was selected for use in an inhouse diffusion processing and analysis pipeline. All 1-month-old subjects in the testing dataset (n = 24) were used for analysis. The pipeline was repeated three times for each subject with different QC methods: motion-corrupted volumes were identified by a human reader and removed (manual QC), identified by the neural network and removed (model QC), or not removed at all (no QC). In brief, the rest of the pipeline is as follows: Raw dMRI data were denoised and corrected for Gibbs ringing using dwidenoise and mrdegibbs from the MRtrix3 toolbox (Tournier et al., 2019). Eddy current and movement corrections were performed using FSL’s eddy tool (Andersson & Sotiropoulos, 2016; Andersson et al., 2016). Lastly, a bias field correction was applied using N4BiasFieldCorrection from the ANTS registration suite (Tustison et al., 2010). The diffusion tensor for each voxel was estimated using the weighted least squares (WLS) method with the DIPY package (Garyfallidis et al., 2014). Quantitative maps of FA, MD, RD, and AD were derived. Additionally, the data were also fit to the three-compartment NODDI model (Zhang et al., 2012) using the Watson distribution and the *brute2fine* optimizer using the Dmipy toolbox (Fick et al., 2019).

Using antsMultivariateTemplateConstruction2.sh, an FA template was created using a subsample of eight high-quality 1-month-old subjects. The NIHPD Objective 2 (Fonov et al., 2011) T2w template for 0 to 2 months of age was used as a reference image, and 10 iterations were run. All FA maps were then registered to template space using symmetric diffeomorphic normalization with ANTs (Avants et al., 2011), and the affine and non-linear warps were saved. Corpus callosum and internal capsule ROIs were obtained from the JHU Neonate Atlas (Oishi et al., 2011). The single subject FA map from the atlas was spatially aligned to the study template using ANTs. The ROIs were then warped into each subject’s native space by first applying the inverse transform of the atlas to the study template, and then the inverse transform of the subject images to the template. Finally, native-space FA, RD, and ICVF maps were masked using the transformed ROIs and nonzero voxel values were extracted. The metrics and ROIs were selected as exemplars for neuroimaging studies of the 1-month brain. The three metrics (FA, RD, and ICVF) were selected for analysis due to their apparent sensitivity to myelination (Dean et al., 2017), and the three regions (corpus callosum and posterior and anterior internal capsules) were selected as early myelinating structures (Dean et al., 2015, 2016; Deoni et al., 2012).

#### 2.4.3 Statistical analyses

Group differences between QC methods were examined for FA, RD, and ICVF values in the corpus callosum (CC) and posterior and anterior internal capsules (pIC, aIC). The concordance correlation coefficient (CCC) (Lin, 1989) was calculated between the gold-standard manual QC method and both the DL model-based QC method and no QC method. The CCC was chosen as a reproducibility index for the methods as other validation techniques such as the Pearson correlation coefficient and paired t-test are not always fully suited for examining reproducibility (Lin, 1989; Morgan & Aban, 2016). Demonstrated low reproducibility between the manual QC and no QC methods will support the need for volume removal in pediatric dMRI preprocessing. It is hypothesized that the proposed DL model-based QC method will demonstrate high reproducibility with the manual QC method, supporting the utility of the neural network for classification. To further demonstrate the need for QC, t-tests and F-tests for variance were run on each individual participant’s ROIs for each metric between the DL model-based QC method and no QC, and it is hypothesized that the mean and/or variance will differ significantly for some participants if motion-corrupted volumes are left in the data.

## 3 Results

### 3.1 Artifact prediction

**Fig. 3.**
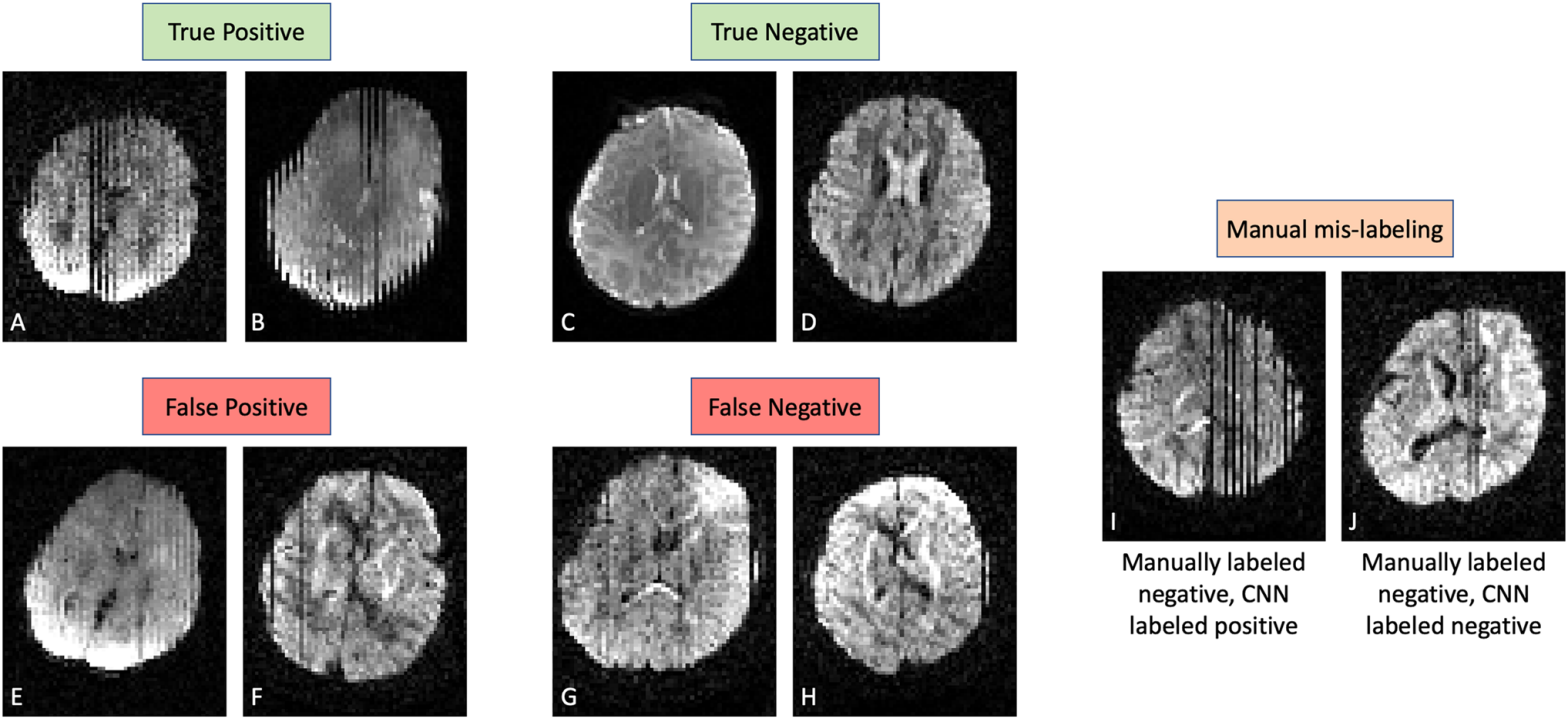
Example dMRI slices from Dataset A are shown below their volume-wise labeling from the model with respect to the manual ground-truth labeling. Panels (A) and (B) are from volumes correctly identified as motion-corrupted, while panels (C) and (D) are from motion-free volumes. Panels (E) and (F) are from volumes flagged for motion by the model but had been manually labeled as motion-free despite containing minor motion artifacts. Inversely, panels (G) and (H) had been manually labeled as motion-corrupted but labeled by the model as motion-free. Lastly, panels (I) and (J) were both manually mis-labeled, although only panel (I) was correctly labeled by the network. Panels (E-J) all represent examples of minor motion (subtle interleaving, single-slice dropout) that can confound both model and manual labeling.

A summary of the network performance for the four cross-validation folds can be found in Table 2. The network achieved a mean accuracy of 95.4% on Dataset A and a mean accuracy of 98.5% on Dataset B. The mean Precision for Datasets A and B was 97.5% and 80.0% respectively, while mean Recall (or True Positive Rate, TPR) and mean True Negative Rate (TNR) were 93.3% and 97.6% respectively for Dataset A, and improved to 95.6% and 98.7% respectively on Dataset B. Figure 3 shows representative labeling on Dataset A from the model trained on fold 1 (Table 2). Of the 612 volumes in the testing set, there were 301 True Negatives, 291 True Positives, 15 False Negatives, and 5 False Positives. On the same GPU as model training (NVIDIA RTX 2070), the mean model evaluation time for an individual diffusion-weighted volume was 75 milliseconds. For a full DWI dataset with 105 direction volumes, the mean model evaluation time is just under 8 seconds.

**Table 2.** Neural network testing results.

	Dataset A				Dataset B			
	Accuracy (%)	Precision (%)	True positive rate (%)	True negative rate (%)	Accuracy (%)	Precision (%)	True positive rate (%)	True negative rate (%)
Fold 1	96.7	98.3	95.1	98.4	98.4	77.9	96.8	98.5
Fold 2	95.3	96.6	94.0	96.6	98.0	74.8	93.5	98.2
Fold 3	96.2	98.6	93.7	98.6	98.8	84.3	95.2	99.0
Fold 4	93.6	96.6	90.6	96.7	98.8	82.8	96.8	98.9
Mean	95.4 ± 1.4	97.5 ± 1.1	93.3 ± 1.9	97.6 ± 1.1	98.5 ± 0.4	80.0 ± 4.4	95.6 ± 1.5	98.7 ± 0.4

Mean and standard deviations were calculated using four networks trained using the cross-validation folds. Dataset A was partitioned for both training and testing the network, while Dataset B was used exclusively for testing.

### 3.2 Quantitative parameter analysis results

**Fig. 4.**
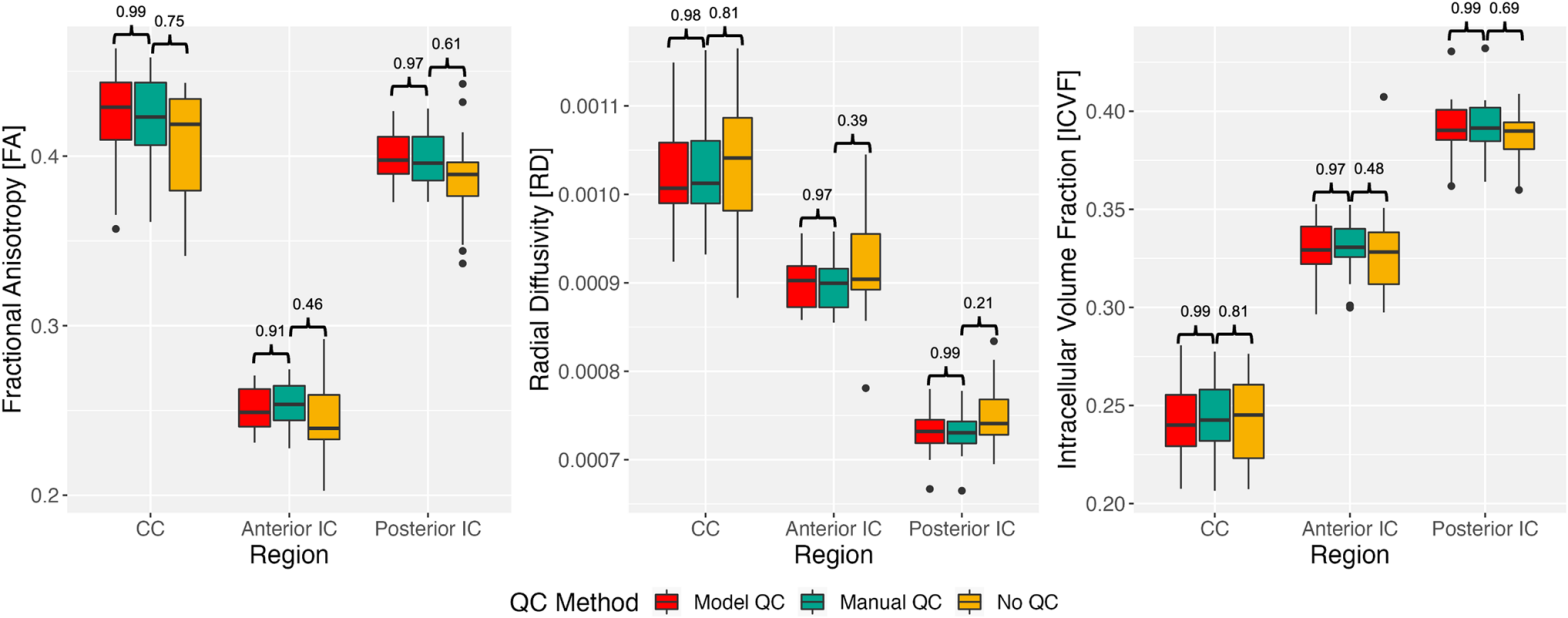
Box plots displaying comparisons between QC processing pipelines for three quantitative measures (FA, RD, ICVF) and three brain regions (corpus callosum, anterior internal capsules, posterior internal capsules) using data from 24 of the 1-month-old participants in Dataset A. The concordance correlation coefficient (CCC) between each QC method is shown above each box plot. A CCC closer to 1 demonstrates high reproducibility.

Group differences between QC methods for the 24 one-month-old subjects in the testing dataset were examined using the CCC. Mean FA, RD, and ICVF values were computed for each participant and box plots for each region, quantitative measure, and QC method are shown in Figure 4. The CCC between the gold-standard manual QC method and the remaining QC methods is shown above each box plot.

The CCC between manual QC and DL model-based QC demonstrates high reproducibility (>0.90) for each metric and brain region. When comparing manual QC and no QC, the CCC demonstrates lower reproducibility (<0.90) for each metric and brain region for the no QC method.

t-tests and F-tests for variance were run on each individual participant’s ROIs and revealed significant differences in both the mean and variance. In 14 of the 24 participants, significant differences that survived multiple comparisons corrections were found between the model QC and no QC methods in the mean of at least one quantitative measure in one region. Table 3 summarizes the t- and F-statistics and denotes significant results for the individual analysis.

**Table 3.** t- and F-test results from each individual participant ROIs comparing the DL model-based QC and no QC processing pipelines for three quantitative measures (FA, RD, ICVF) and three brain regions (corpus callosum, anterior internal capsules, posterior internal capsules).

Subj	FA						RD						ICVF					
	CC		Ant. IC		Post. IC		CC		Ant. IC		Post. IC		CC		Ant. IC		Post. IC	
	T-Stat	F-Stat	T-Stat	F-Stat	T-Stat	F-Stat	T-Stat	F-Stat	T-Stat	F-Stat	T-Stat	F-Stat	T-Stat	F-Stat	T-Stat	F-Stat	T-Stat	F-Stat
1	0.08	1.06	0.24	1.04	0.89	1.06	0.58	1.16	0.15	1.18	−1.12	1.04	−0.34	1.03	−0.63	1.27	1.03	1.03
2	3.94*	1.11	2.75*	1.18	2.77*	0.95	−2.29	1.08	−3.81*	1.04	−2.93*	0.98	3.18*	1.16	4.12*	0.97	3.54*	1.09
3	1.38	1.08	−0.57	1.02	−0.65	1.01	−1.60	0.89	2.07	1.08	1.45	1.02	0.77	1.00	−5.16*	0.98	−3.20*	1.11
4	−0.15	1.13	−0.50	1.04	0.56	1.00	−1.10	0.98	0.88	1.16	−0.77	1.05	−0.51	1.09	−2.96*	1.31	−0.29	1.11
5	3.28*	1.17	2.04	1.28	4.80*	1.20	−0.24	1.38	−4.97*	1.00	−5.73*	1.24	1.19	1.16	6.41*	1.00	4.99*	1.18
6	2.02	1.05	1.21	1.13	1.85	1.11	−0.84	0.95	−0.55	1.06	−1.32	1.03	−0.35	1.01	−2.27	0.95	−1.80	0.97
7	3.45*	1.11	2.49*	1.22	3.05*	1.11	−1.50	1.03	−1.80	1.15	−2.81*	1.12	2.17	1.08	0.37	1.23	2.35	1.12
8	2.18	1.31	−4.75*	0.75	−1.74	0.66*	−4.16*	1.90	−14.33*	0.34*	−18.63*	0.83	−1.68	1.13	11.01*	0.23*	6.91*	0.33*
9	−2.28	0.84	−2.44*	1.04	−2.04	1.24	6.70	0.85*	8.43*	0.93	0.60	1.26	−7.63*	0.50*	−11.31*	0.18*	0.37	0.79*
10	0.54	0.95	0.24	0.95	−0.01	1.01	1.07	1.18	−0.11	0.85	1.31	1.08	−0.78	1.05	0.28	0.92	−1.36	1.09
11	4.95*	1.01	1.86	1.32	1.85	1.08	−3.42*	0.91	−5.18*	0.93	−2.69*	1.04	2.43	1.07	4.75*	0.89	1.55	1.10
12	8.70*	1.25	2.42*	1.23	3.68*	1.30	−3.63*	1.13	−2.55*	1.18	−1.68	0.90	1.68	1.06	−0.87	0.90	−3.87*	0.56*
13	0.87	0.95	0.78	1.10	0.45	1.06	−0.60	0.95	−1.01	0.98	−0.31	1.03	0.61	1.01	0.35	0.86	0.27	1.02
14	1.10	1.11	1.93	1.21	0.72	1.16	−0.94	1.10	−1.64	1.10	−2.13	1.26	−0.32	1.02	−0.78	0.90	2.43*	1.55
15	1.21	1.00	0.02	1.03	0.79	0.98	−0.71	0.88	0.42	1.08	−0.58	0.97	0.04	0.94	−0.79	1.13	−0.33	0.95
16	1.87	1.07	1.03	1.00	1.43	1.01	−0.01	1.24	−0.50	0.98	0.10	1.09	−0.27	1.21	−3.46*	1.01	−3.19*	1.38
17	7.02*	1.37	3.51*	1.47	8.03*	1.40	−4.23*	1.13	−5.26*	1.28	−9.32*	1.22	5.58*	1.37	7.30*	1.28	7.78*	1.13
18	1.36	1.02	0.23	1.04	0.36	1.02	−0.46	1.01	−0.15	1.04	−0.35	1.03	−0.34	1.01	−1.04	1.05	−0.49	1.11
19	0.87	1.04	0.01	1.05	0.26	0.99	−0.33	1.03	−0.45	0.99	−0.12	1.02	0.09	1.03	0.82	0.94	−0.37	1.08
20	2.36	1.02	1.69	1.15	2.64*	1.02	−0.43	0.99	−0.92	1.12	−1.81	0.97	1.01	1.02	0.92	0.98	1.29	1.00
21	4.51*	1.17	2.67*	1.34	6.67*	1.13	−0.85	1.24	−2.23	0.99	−7.75*	1.06	0.07	1.20	−1.45	0.74*	3.41*	1.05
22	−0.12	0.97	0.61	1.09	0.71	1.04	1.43	1.02	−0.10	1.07	0.11	1.17	−1.46	0.86	−0.25	1.09	−0.69	1.27
23	0.54	0.98	0.42	1.07	0.50	1.03	0.13	1.05	−0.95	1.06	−0.83	1.02	−0.31	1.01	1.07	1.05	0.94	1.01
24	0.38	1.08	0.54	1.00	0.61	0.97	−0.30	1.00	−0.64	0.95	−0.38	0.97	0.15	1.00	−0.77	0.89	−0.71	0.98

Significance was defined as p < 0.0167 (bold*) following Bonferroni corrections for multiple comparisons.

## 4 Discussion

### 4.1 Overview

We proposed a three-dimensional CNN to automatically detect motion artifacts in diffusion MRI data spanning the early developmental period from 1 month to 35 months, an age range previously unexplored in prior DL-based dMRI QC work. We trained the network on data acquired at 1 month and 24 months of age and evaluated the network on a second dataset containing data acquired from 2 to 35 months of age using a different diffusion protocol. Our network performance results demonstrate the model’s generalizability to multiple b-value acquisition protocols, different brain sizes, and differing image contrasts during early developmental timepoints. Additionally, results of quantitative parameter estimation without QC, with manual QC, and with our DL network demonstrate the need for and importance of QC of pediatric dMRI and high reproducibility between manual QC and DL-based QC.

### 4.2 Architecture and training datasets

The major challenges with training a neural network for medical imaging tasks typically involve a lack of adequate annotated training data. As discussed earlier, a 3D network was chosen to alleviate the task of manual labeling, requiring manual quality control at the volume level (69 to 105 diffusion volumes per subject) rather than slice level (4,140-7,560 slices per subject). This significantly reduces the time required to QC each subject but moving from 2D to 3D also reduces the amount of data available to train the neural network. Smaller training datasets can make achieving convergence more difficult, due to overfitting and unequal data distributions. With the proposed architecture and hyperparameters, an acceptable mean accuracy of 95% was achieved by training on a moderate dataset (Dataset A) of 2,300 dMRI volumes. An additional challenge with neural networks is generalizability. It can be nontrivial to train a model that generalizes to novel data with acceptable performance. In the case of pediatric diffusion data, novel data may include different acquisition parameters such as diffusion directions and b-values, and subject differences such as brain size, contrast, and pathology. To address concerns of generalization, an unseen dataset (Dataset B) was used to further evaluate the model and achieved an accuracy of 98%.

### 4.3 Analysis of artifact prediction and quantitative fitting performance

Relative to the manually labeled ground truth dataset, the model achieves high accuracy (>90%) across the four folds, with mean accuracies of 95.4% and 98.5% on Datasets A and B, respectively (Table 2). Our model was able to generalize well to Dataset B, which had differences in age (affecting brain volume and contrasts) and acquisition parameters (TR, TE, b-value), despite remaining completely unseen during the training process. The performance differences between the datasets could be due to labeling mistakes and inter-rater discrepancies during the manual QC process, as Dataset B was labeled by one reviewer in a single session while Dataset A was labeled by multiple reviewers over a longer period. Additionally, these performance accuracies were not adjusted to reflect mistakes in the manual labeling process that were discovered after the model had been trained. For example, Figure 3I shows one of three volumes containing severe slice dropout from motion that were missed during manual labeling. In fact, the majority of false positives and false negatives in Dataset A (e.g., Fig. 3E, F, G, H) were minor motion artifacts such as subtle interleaving or single-slice dropout that were manually labeled differently between volumes, potentially due to intra- or inter-rater variability. The most noticeable difference between the performance on the datasets is precision, which dropped from 97.5% to 80.0% between Datasets A and B. Low precision is driven by a large number of false positives with respect to true positives. A brief retrospective analysis of the Dataset B model-based labeling revealed that nearly all false positive labels were due to very subtle interleaving and dropout that had been deemed insignificant by the manual labeler. However, the predicted value for these volumes was close to the default decision threshold of 0.5. As discussed in Graham et al. (2018) and Ahmad et al. (2023), the decision threshold can be reduced to increase artifact sensitivity and increased to reduce sensitivity to minor artifacts. Ultimately, the severity of motion artifacts deemed unacceptable is a subjective decision made by researchers, and it is unlikely that a single automated QC tool such as the proposed work uses artifact criterion that all researchers agree upon. Thus, it is recommended that researchers manually annotate and process a small number of cases with motion-corrupted volumes and determine an appropriate decision threshold for the trained model before processing an entire dataset. Additionally, researchers can use the model output to identify borderline cases with a probability close to the decision threshold and decide whether to raise or lower the threshold to include or exclude such cases.

Figure 4 presents the group differences between QC methods and the lack of QC on a selection of DTI and NODDI parameters in three exemplar brain regions for pediatric development research. For all metrics and brain region combinations, our reproducibility index, the CCC, was higher between the gold-standard manual QC and the DL model-based QC methods (>0.90) and lower between manual QC and no QC (≤0.81). This high reproducibility for DTI and NODDI parameters between the methods supports the utility of the model to replace or augment manual QC in standard dMRI pre-processing pipelines without a significant impact on quantitative results.

Individual differences between the DL model-based QC methods and no QC were examined using t- and F-tests, with results shown in Table 3. Significant differences in the mean that survived multiple comparisons corrections were found in 14 of the 24 participants for at least one quantitative measure in one region. These results demonstrate the effect of motion on individual results and the importance of QC in dMRI pre-processing pipelines.

### 4.4 Comparison to other automated deep-learning-based QC tools

Two prior studies (Graham et al., 2018; Kelly et al., 2017) trained and tested exclusively on neonates, and generalizability to datasets including older children was not examined. Samani et al. (2020) demonstrated that a trained artifact QC model (QC-Automator) could generalize well to previously unseen datasets with different acquisition protocols, although with accuracy losses up to 14%. However, the effect of different pathologies and ages was not investigated. In a study from the same group with a new 3D network (3D-QCNet; Ahmad et al., 2023), the network was trained on three datasets and tested on four unseen datasets with varying acquisition protocols, pathologies, and age ranges. However, none of the seven datasets contained participants under the age of 6, and the lowest accuracy and recall were observed on the datasets with adolescents and young adults (6-25 years). Thus, it is unlikely that previously developed models such as QC-Automator and 3D-QCNet would generalize well to infant data without additional fine-tuning.

To our knowledge, our method describes the first automated QC technique trained and validated on early pediatric dMRI that is not limited to a narrow time point (e.g., neonates) or starts at school age (e.g., 6 years and older). Due to the differences in ages and how motion may manifest differently between asleep and awake exams, it is uncertain whether prior deep learning-based automated QC approaches could generalize well to this early pediatric age range. Thus, the proposed method fills this gap left by prior studies.

### 4.5 Limitations and future work

We have shown that our proposed method can be used for rapid and accurate automated motion artifact detection for early pediatric multi-shell diffusion data. However, there are several potential limitations that may affect further widespread adoption. As mentioned by Ettehadi et al. (2022), many prior approaches for automated dMRI QC (including this work) perform binary QC, typically classifying exclusively motion or assigning “poor” or “good” labels. To perform truly automated QC, all possible artifacts must be detected and correctly classified, such as FOV truncation errors (Ettehadi et al., 2022). While FOV truncation is not a common artifact in the 1-month-old participants in Dataset A or the infants and toddlers in Dataset B, it becomes more common at toddler age and above when the FOV is kept small to reduce scan time, but the child shifts and moves out of the FOV mid-scan. In our limited sample and age range of pediatric data all sourced from a single scanner model, motion was the dominant artifact and the sole focus of training due to the absence of other artifacts. Additionally, both Datasets A and B contain only data from typically developing infants, with no clinically relevant pathology such as lesions from injury. It is expected that with the inclusion of clinically relevant pathology in the training dataset, a re-trained network would be agnostic to pathology (Ahmad et al., 2023). With access to more data across a wider age range and a greater variety of artifacts, an all-purpose automated QC tool could be trained for use across the pediatric years. In the future, we plan to expand our training dataset using publicly available datasets such as the Baby Connectome Project (BCP) and the Healthy Brain Network (HBN) to create a dataset that spans the early development years and includes a wider variety of artifacts, scanners, and acquisition protocols. Additionally, the wider variety of artifacts present will be leveraged to perform multi-label classification to create an automated QC method with broader utility.

In recent years, the number of studies investigating early brain development has increased due to advances in both imaging techniques and best practices for scanning sleeping children. Several of these are large-scale, such as the Developing Human Connectome Project (dHCP, https://www.developingconnectome.org/; Edwards et al., 2022), Baby Connectome Project (BCP, https://babyconnectomeproject.org/; Howell et al., 2019), and the upcoming Healthy Brain and Child Development (HBCD) study (https://hbcdstudy.org/; Jordan et al., 2020). For large studies such as these, manual QC would be overly time-consuming, require an extensive team of expert raters whose inter-rater reliability would need to be measured and compared. dHCP and BCP have implemented automated QC methods (i.e., DTIPrep, EDDY) as alternatives to manual QC. However, existing automated QC methods may not consistently perform well on infant brains. Additionally, if quantitative measures are going to be extracted from the data and used to characterize development, it is important to have reliable and reproducible measures and the effect of automated QC methods (i.e., interpolation, registration) on these measures is not always investigated. Meanwhile, the effect of manual volume removal on DTI measures has been investigated, albeit with a smaller number of directions (Chen et al., 2015).

As a method trained and validated on subjects between 1 and 35 months of age, the proposed method can be further modified and implemented for the ongoing HBCD study, which seeks to scan 7,500 participants across the first 10 years of life. The results of evaluation on Dataset B suggest the proposed method may generalize well to other datasets with differing diffusion protocols, although re-training or fine tuning the network may be necessary for optimal performance.

## 5 Conclusion

Motion artifact detection is a necessary but time-consuming and error-prone step in the pipeline for processing diffusion MRI data, especially when scanning pediatric participants where motion may be severe and frequently occurring. Moreover, dMRI models, such as DTI and NODDI, may be confounded by motion-related artifacts, resulting in an important need to identify and account for motion-corrupted volumes prior to further analyses. In this work, we proposed a 3D CNN to perform automated motion artifact detection in early pediatric dMRI data and replace manual QC. Our results demonstrate high accuracy on both a training dataset (95%) and an unseen dataset (98%), which indicates generalizability. Furthermore, we showed that DL-based QC results demonstrate high reproducibility for DTI and NODDI metrics compared to the gold-standard manual QC. To the best of our knowledge, this is the first dMRI QC CNN to utilize early pediatric data in training and could be integrated in dMRI processing pipelines for future studies of early brain development.

## Data Availability

The source code, trained model, and instruction files for this work are provided in a repository at https://github.com/jmweaver-uw/infQC_pub. The authors welcome requests for additional information or assistance regarding the code and model.
